# Blood–Nerve Barrier Breakdown Induced by Immunoglobulin G in Typical and Multifocal Chronic Inflammatory Demyelinating Polyneuropathy and Multifocal Motor Neuropathy

**DOI:** 10.3390/ijms27021088

**Published:** 2026-01-22

**Authors:** Fumitaka Shimizu, Ryota Sato, Yoichi Mizukami, Kenji Watanabe, Toshihiko Maeda, Takashi Kanda, Naoko Matsui, Sonoko Misawa, Yuishin Izumi, Satoshi Kuwabara, Masayuki Nakamori

**Affiliations:** 1 Department of Neurology and Clinical Neuroscience, Yamaguchi University Graduate School of Medicine, Ube 755-8505, Japanmnakamor@yamaguchi-u.ac.jp (M.N.); 2 Center for Gene Research, Yamaguchi University, Ube 755-8505, Japan; mizukami@yamaguchi-u.ac.jp (Y.M.);; 3 Department of Neurology, Tokushima University Graduate School of Biomedical Sciences, Tokushima 770-8503, Japan; 4 Department of Neurology, Graduate School of Medicine, Chiba University, Chiba 260-8670, Japan; 5 Department of Neurology and Neurological Science, Institute of Science, Tokyo 113-8519, Japan

**Keywords:** blood-nerve barrier, chronic inflammatory demyelinating polyneuropathy, multifocal motor neuropathy, immunoglobulin G

## Abstract

Impairment of the blood–nerve barrier (BNB) is associated with the pathogenesis of chronic inflammatory demyelinating polyneuropathy (CIDP) and multifocal motor neuropathy (MMN). This research analyzes the molecular mechanisms of immunoglobulin (Ig) G in patients with typical CIDP, CIDP variants (multifocal CIDP), and multifocal motor neuropathy in BNB-endothelial cells. IgG was purified from the sera of patients with typical CIDP (n = 15), multifocal CIDP (n = 14), multifocal motor neuropathy (MMN; n = 12), and healthy controls (HCs; n = 14). Molecular changes in the RNA-seq/high-content imaging system and permeability were evaluated after the incubation of human peripheral nerve microvascular endothelial cells (PnMECs) with IgG. RNA-seq and a pathway analysis using PnMECs showed that TNF-α, CCL20 (MIP-3α), and ICAM-1 were the centers of the upregulated gene pathways in patients with typical CIDP. TNF-α, VCAM-1, NF-κB, and CSF2 (GM-CSF) are important molecules in patients with multifocal CIDP. The high-content imaging system demonstrated that MIP-3, GM-CSF, and VCAM-1 increased after exposure to typical CIDP-IgG, claudin-5 decreased after exposure to IgG from patients with multifocal CIDP, and TNF-α and VCAM-1 increased after exposure to IgG from patients with MMN. The 10 kDa dextran permeability using coculture with PnMECs and pericytes increased after exposure to IgG from patients with typical CIDP and multifocal CIDP. This effect was reversed after incubation with GM-CSF neutralizing antibodies. Upregulation of MIP-3, GM-CSF, and VCAM-1 may contribute to the infiltration of leukocytes/lymphocytes/monocytes across the BNB into the PNS in typical CIDP. IgG from typical CIDP and multifocal CIDP may decrease barrier properties through autocrine GM-CSF from PnMECs. VCAM-1 upregulation through autocrine TNF secretion in PnMECs may induce lymphocyte entry across the BNB in MMN.

## 1. Introduction

Chronic inflammatory demyelinating polyneuropathy (CIDP) is an immune-mediated and treatable neuropathy that responds to immunomodulatory therapies, including steroids, intravenous immunoglobulin (IVIg), and plasmapheresis [1]. The immunopathogenesis of CIDP is still unclear; however, B cells, autoantibodies, and T cells seem to play an important role in their pathogenesis, as treatment against soluble factors, including IVIg, PE, and FcRn inhibitors, was effective for CIDP [2,3,4]. Clinically, CIDP is classified into typical CIDP and CIDP variants, including multifocal CIDP, distal CIDP, motor CIDP, and sensory CIDP, based on the 2021 diagnostic guidelines for EAN/PNS [5]. Typical CIDP is defined as symmetric motor/sensory symptoms with proximal and distal weakness, but multifocal CIDP is characterized by upper-limb dominant asymmetric motor/sensory symptoms, suggesting multiple mononeuropathies with conduction blocks (CBs) [5]. The difference in these clinical features between typical CIDP and multifocal CIDP is based on a different immunopathogenesis between the two phenotypes: a hypothesis that humoral immunity may be associated with typical CIDP, but cellular immunity may be related to multifocal CIDP has been suggested [6]. MMN is clinically characterized by upper-limb dominant asymmetric motor symptoms with CBs and is distinguished from multifocal CIDP by the ineffectiveness of steroid therapy [7,8]. The immunopathomechanism of MMN is still unknown, but humoral immunity is involved, as IVIg treatment is effective [7,8].

Breakdown of the blood–nerve barrier (BNB) is a key feature of the pathogenesis of CIDP and MMN [9,10,11]. The BNB is formed by endothelial cells and pericytes in endoneurial microvessels and acts as a barrier between the blood and the peripheral nervous system [12]. Pathological findings demonstrated a decrease in tight junctions in the sural nerve from typical CIDP and perivascular lymphocytic accumulation around the BNB in motor nerves from MMN [13,14]. Our previous studies demonstrated that sera from patients with CIDP or MMN showed a reduction in the tight junction protein claudin-5 and in the barrier function, and sera from patients with multifocal CIDP and MMN showed increased IP-10 in human BNB-endothelial cells [9,10,11]. In the present study, we investigated the effects of serum IgG on human BNB-endothelial cells in individual patients with typical CIDP, multifocal CIDP, and MMN using RNA-seq, high-content imaging, and permeability assays.

## 2. Results

### 2.1. Identification of the Changed Gene Expression in FH-BNB Cells After Exposure to IgG from Typical CIDP, Multifocal CIDP, or MMN Patients by RNA-seq

**Figure 1:**
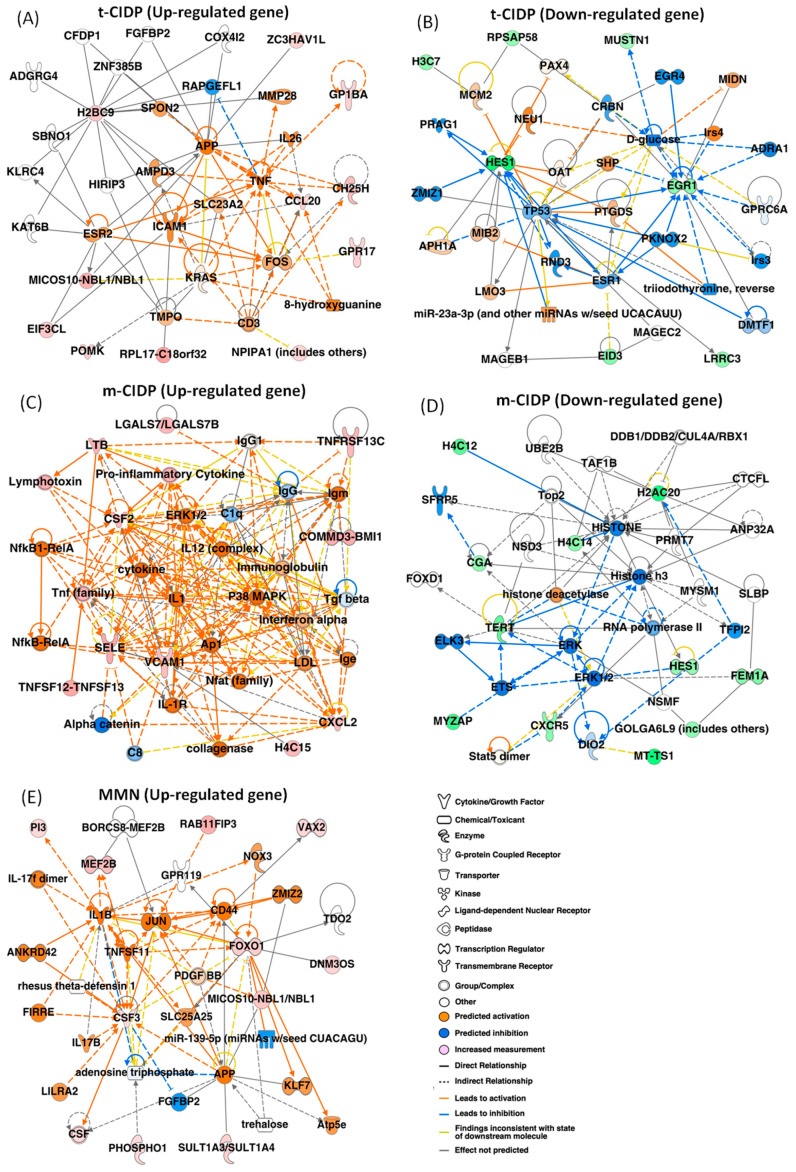
A whole transcriptome analysis with RNA-seq of PnMECs after exposure to IgG from patients with typical CIDP, multifocal CIDP, and MMN. PnMECs from patients with typical CIDP (n = 4), multifocal CIDP (n = 4), MMN (n = 4), and healthy controls (n = 4) were incubated with IgG. PnMECs not exposed to IgG were used as controls. More than 57,000 genes were detected in approximately 23–30 million reads in each sample. In the network analysis of upregulated genes, chemokine (C-C motif) ligand 20 (CCL20), TNF-α, and ICAM-1 were detected as upregulated genes (**A**), and HES-1 and EGR-1 were detected as downregulated genes (**B**) in the center of the network analysis in t-CIDP patients. CSF2 and VCAM-1 were detected as upregulated genes (**C**) and HES-1 was detected as a downregulated gene (**D**) in multifocal CIDP patients, and CSF3 and FOXO1 were detected as upregulated genes in MMN patients (**E**) in the center of the network analysis. The red nodes show the upregulated genes, and the green nodes indicate the downregulated genes in the RNA-seq analysis (FC > 1.5; *p* < 0.05).

IgG from typical CIDP (n = 4), multifocal CIDP (n = 4), and MMN patients (n = 4) who showed the same clinical phenotype at the last follow-up examination was selected for RNA-seq. IgG from HCs (n = 4) was used as the controls. A whole-transcriptome analysis by RNA-seq in FH-BNB cells was performed after exposure to patient/HC IgG. More than 57,000 genes were detected in approximately 23–30 million reads in each sample. Heat maps at the same *p* values and fold change (FC) showed that 198 genes (124 upregulated genes and 74 downregulated genes) between typical CIDP patients and HCs, 588 genes (329 upregulated genes and 259 downregulated genes) between multifocal CIDP patients and HCs, and 183 genes (123 upregulated genes and 60 downregulated genes) between MMN patients and HCs were significantly differentially expressed (FC > 1.5; *p* < 0.05) (Supplemental Material, Supplemental Figures S1–S4). In the Ingenuity Pathway Analysis (IPA), chemokine (C-C motif) ligand 20 (CCL20)/MIP-3α, TNF-α, and ICAM-1 as the upregulated genes (Figure 1A) and HES-1 and EGR-1 as the downregulated genes (Figure 1B) were detected in the center of the network analysis in typical CIDP patients. CSF2/GM-CSF and VCAM-1 as upregulated genes (Figure 1C) and HES-1 as downregulated genes (Figure 1D) in multifocal CIDP patients and CSF3/G-CSF and FOXO1 as upregulated genes in MMN patients (Figure 1E) were detected in the center of the network analysis.

### 2.2. Change in MIP-3α, Claudin-5, GM-CSF, VCAM-1, TNF-α, ICAM-1, and IP-10 in BNB-Endothelial Cells After Exposure to Patient IgG

**Figure 2:**
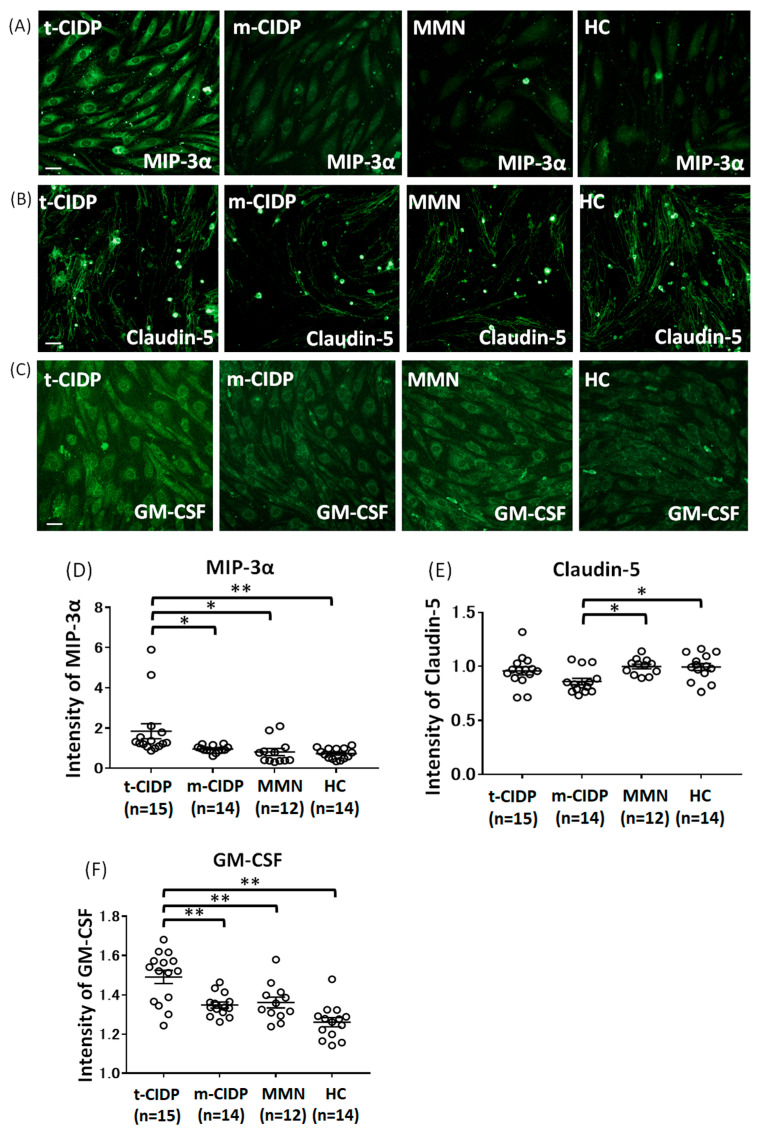
Changes in the MIP3-α, claudin-5, and GM-CSF after exposure to IgG from patients with typical CIDP, multifocal CIDP, and MMN. Immunostaining of human peripheral nerve microvascular endothelial cells (PnMECs) for MIP3-α (**A**), claudin-5 (**B**), and GM-CSF (**C**) (green) after exposure to IgG (500 µg/mL) from patients with typical CIDP, multifocal CIDP, and MMN or healthy controls (HC). Images were captured using In Cell Analyzer 2000. Scale bar, 50 μm. Scatter plots of the intensities of MIP3-α (**D**), claudin-5 (**E**), and GM-CSF (**F**) in PnMECs, as determined by high-content imaging after exposure to IgG from patients with typical CIDP (n = 15), multifocal CIDP (n = 14), MMN (n = 12), and healthy controls (HCs; n = 14). The data were normalized to cultures that had not been exposed to human IgG, and are shown from three independent experiments. The *p* values were determined using an unpaired Student’s *t*-test (two-sided) (* *p* < 0.05, ** *p* < 0.01 vs. the HC group).

**Figure 3:**
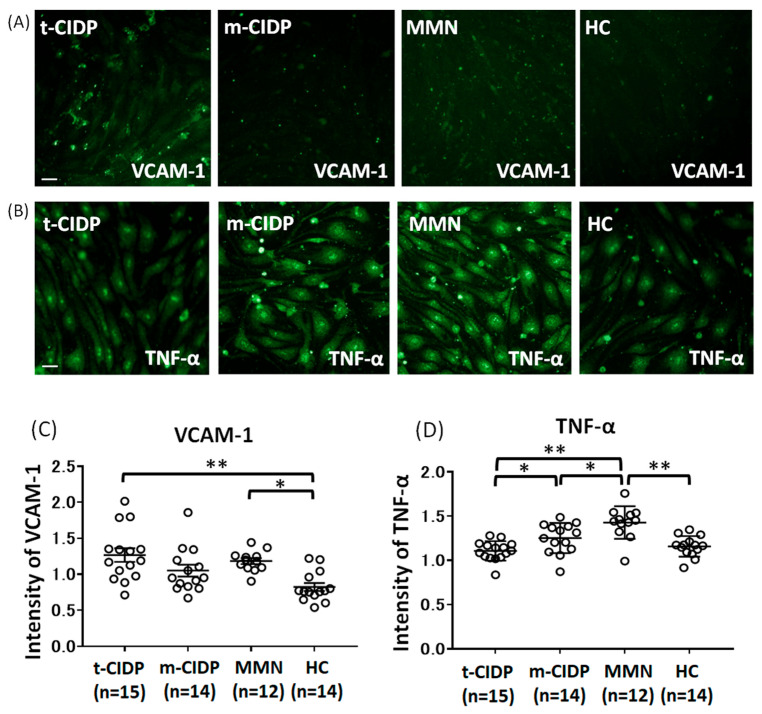
Changes in the VCAM-1 and TNF-α after exposure to IgG from patients with typical CIDP, multifocal CIDP, and MMN. Immunostaining of PnMECs for VCAM-1 (**A**) and TNF-α (**B**) (green) after exposure to IgG (500 µg/mL) from patients with typical CIDP, multifocal CIDP, and MMN or healthy controls (HC). Images were captured using In Cell Analyzer 2000. Scale bar, 50 μm. Scatter plots of the intensities of VCAM-1 (**C**) and TNF-α (**D**) in PnMECs, as determined by high-content imaging after exposure to IgG from patients with typical CIDP (n = 15), multifocal CIDP (n = 14), MMN (n = 12), and healthy controls (HCs; n = 14). The data were normalized to cultures that had not been exposed to human IgG and are shown from three independent experiments. The *p* values were determined using an unpaired Student’s *t*-test (two-sided) (* *p* < 0.05, ** *p* < 0.01, vs. the HC group).

We selected the CCL20/Macrophage Inflammatory Protein-3 (MIP-3α), TNF-α, ICAM-1, CSF2/GM-CSF, VCAM-1, and TNF-α as the upregulated genes for an immunohistochemistry, which are detected in the center of the network analysis using RNA-seq and pathway analysis. We have also chosen IP-10 and claudin-5, as our previous studies demonstrated that changes in these molecules are associated with the breakdown of the BNB after exposure to sera from CIDP patients [9,10]. Immunohistochemistry and high-content imaging were applied after incubation with IgG from patients with typical CIDP (n = 15), multifocal CIDP (n = 14), MMN (n = 12), and HCs (n = 14) to evaluate the changes in the amounts of proteins, including CCL20/MIP-3α, claudin-5, CSF2/GM-CSF, VCAM-1, TNF-α, ICAM-1, and IP-10 (Figure 2A–F and Figure 3A–D). The amounts of MIP-3α and GM-CSF were significantly increased after exposure to IgG from patients with typical CIDP in comparison to those from multifocal CIDP, MMN, and HCs (Figure 2A,C,D,F). The amount of claudin-5 in the multifocal CIDP group was significantly lower than that in the MMN and HCs groups (Figure 2B,E). The expression of VCAM-1 was significantly increased after incubation with IgG from typical CIDP and MMN in comparison to that from HCs (Figure 3A,C). The amount of TNF-α was significantly increased after exposure to IgG from MMN in comparison to that from typical CIDP, multifocal CIDP, and HCs, and was also significantly higher in the multifocal CIDP group than that in the typical CIDP group (Figure 3B,D). The expression of ICAM-1 and IP-10 was not significantly changed after exposure to IgG from typical CIDP, multifocal CIDP, or MMN patients or HCs (Supplemental Figure S5).

### 2.3. Change of 10 kDa-Dextran Permeability in BNB-Endothelial Cells After Exposure to IgG from Typical CIDP, Multifocal CIDP, and MMN Patients and HCs

**Figure 4:**
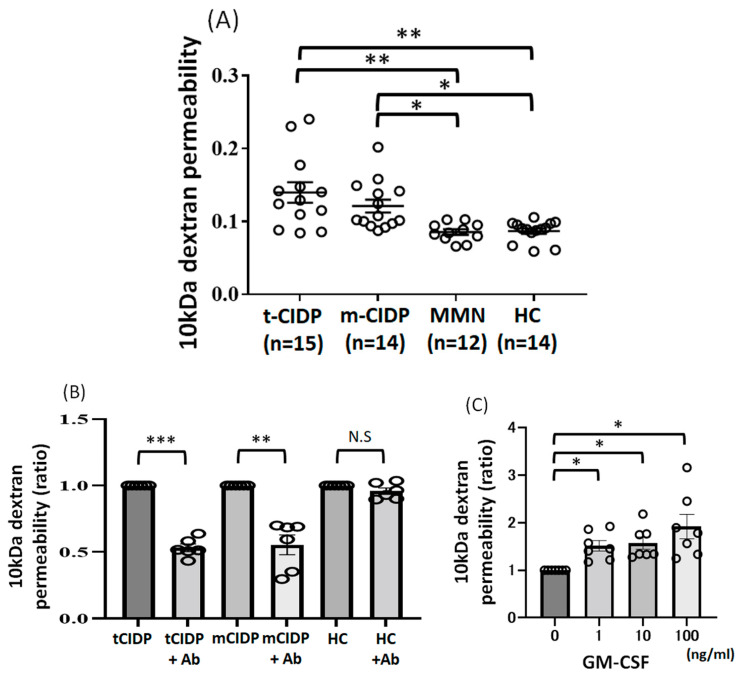
Changes in 10 kDa-dextran permeability in PnMECs after exposure to IgG from each patient and the effect of blocking GM-CSF on the permeability after incubation of IgG from each patient in PnMECs. (**A**) The change in 10 kDa-dextran permeability coefficient in PnMECs was determined after exposure to IgG (500 µg/mL) from patients with typical CIDP (n = 15), multifocal CIDP (n = 14), MMN (n = 14), and healthy controls (n = 14). The *p* values were determined by Tukey’s multiple comparison test. (**B**) The permeability of 10 kDa dextran was evaluated after incubation with IgG from typical CIDP, multifocal CIDP, and healthy controls with or without blocking of GM-CSF using anti-GM-CSF neutralizing antibodies in the coculture BNB model. (**C**) The effect of incubation with GM-CSF on the increase of 10 kDa permeability in FH-BNB. (* *p* < 0.05, ** *p* < 0.01, *** *p* < 0.001, N.S, not significant).

We measured the permeability of 10 kDa-dextran in a monolayer coculture BNB model consisting of FH-BNB cells and pericytes after exposure to IgG from patients with t-CIDP (n = 15), multifocal CIDP (n = 14), MMN (n = 12), and HCs (n = 14). The 10 k-Da permeability was significantly elevated after exposure to IgG from patients with typical CIDP or multifocal CIDP in comparison to IgG from patients with MMN and HCs (Figure 4A).

Blocking GM-CSF using anti-GM-CSF neutralizing antibodies significantly decreased the permeability of 10 kDa dextran after incubation with IgG from typical CIDP or multifocal CIDP (Figure 4B) in a coculture BNB model. This effect was not observed after incubation with IgG from HCs (Figure 4B). Incubation of FH-BNB with GM-CSF significantly increased 10 kDa permeability (Figure 4C).

## 3. Discussion

Disease-specific autoantibodies against t-CIDP, multifocal CIDP, and MMN have not yet been identified, although autoantibodies against nodal and paranodal proteins, including contactin-1, neurofascin (NF) 155, nodal NF140/186, and contactin-associated protein-1, have recently been identified in a small fraction of CIDP patients [15]. Previous neurophysiological studies have suggested that passive transfer of IgG or sera from CIDP patients to experimental animals induces conduction block, demyelination, and reduction in motor nerve conduction velocity, suggesting that antibody-mediated mechanisms contribute to the demyelinating process [16,17]. A recent clinical trial, the ADHERE Study, demonstrated that efgartigimod-alpha, a human IgG1 antibody Fc fragment (Neonatal Fc receptor antagonist), has the effect of reducing the risk of relapse in patients with CIDP who responded to treatment versus placebo [18]. Efgartigimod reduces the binding of endogenous IgG, including pathogenic autoantibodies, to FcRn, resulting in decreased levels of serum IgG. These data suggest that the reduction in serum IgG using FcRn inhibitors leads to a therapeutic effect in CIDP, and antibody-mediated mechanisms may contribute to the pathogenesis of CIDP [18]. However, the detailed molecular mechanism of the IgG responsible for the development of the disease in patients with CIDP remains elusive.

In the present study, we evaluated the effect of IgG from patients with typical CIDP, multifocal CIDP, and MMN on the blood–nerve barrier at the molecular level using the human BNB-endothelial cell line (PnMECs). RNA-seq and a pathway analysis demonstrated that TNF-α, CCL20 (MIP-3α), and ICAM-1 in typical CIDP patients and TNF-α, VCAM-1, NF-κB, and CSF2 (GM-CSF) in multifocal CIDP patients were important upregulated molecules. An evaluation at the protein level using a high-content imaging system demonstrated an increase in MIP-3, GM-CSF, and VCAM-1 in the typical CIDP group, a reduction in claudin-5 in the multifocal CIDP group, and an elevation of TNF-α and VCAM-1 in the MMN group as the important molecules. The permeability of the PnMEC monoculture increased after exposure to IgG from typical CIDP or multifocal CIDP patients, in comparison to that from healthy controls. Incubation with GM-CSF enhanced the barrier function, and the inhibition of GM-CSF using neutralizing GM-CSF antibodies reversed this effect in typical CIDP or multifocal CIDP groups, suggesting that the autocrine secretion of GM-CSF induced the increased permeability of BNB-endothelial cells.

CCL20 (MIP-3α), the only high-affinity chemokine ligand for C-C motif chemokine ligand receptor 6 (CCR6), is a mediator deeply involved in CCR6+ leukocyte migration during inflammation [19]. CCR6 is expressed in several cells, including B cells, regulatory CD4 T cells, Th17 cells, immature dendritic cells, and innate lymphoid cells (ILCs) [19]. CCL20 is secreted mainly by epithelial and endothelial cells, and its expression is increased by the stimulation of several inflammatory cytokines, including IL-1α, IL-1β, IL-17, IL-21, TNF-α, and IFN-γ [19,20]. In addition, IFN-γ- and GM-CSF-secreting T cells expressing CCR6 are enriched in the CSF of patients with multiple sclerosis (MS), probably playing a pathogenic role in this disease [21]. However, the pathogenicity of CCR6-expressing T cells in CIDP or MMN is unknown. The present study demonstrated that the CCL20 expression in PnMECs was increased after exposure to IgG from typical CIDP patients, suggesting that stimulation with an unknown autoantibody, including typical CIDP-IgG, induces CCL20 secretion from BNB-endothelial cells and drives the migration of CCR6-expressing lymphocytes to BNB-endothelial cells, resulting in the penetration of pathogenic CCR6-expressing lymphocytes across the BNB.

The hematopoietic growth factor GM-CSF is produced by several cells, including T cells, macrophages, endothelial cells, and fibroblasts, in response to immune activation, and is expressed on the surface of cells [22,23]. GM-CSF plays an important role in recruiting circulating neutrophils, lymphocytes, and monocytes to increase their resistance to local infection [22,23]. In experimental autoimmune encephalomyelitis (EAE), GM-CSF is released by endothelial cells in the blood–brain barrier after stimulation with IL-1β, thus stimulating macrophages to release more IL-1β [22,23]. Furthermore, GM-CSF-producing CD4+ T cells, CD8+, Th17, and B cells are involved in the pathogenesis of MS [23]. Some studies have demonstrated that GM-CSF increases the permeability of small molecules through the decrease in claudin-5 and ZO-1 and increases the transmigration of lymphocytes via an increase in CCL2 in the blood–brain barrier [24,25]. The present study showed that GM-CSF is secreted by PnMECs after exposure to IgG from patients with t-CIDP, which may stimulate the migration of GM-CSF-producing T or B cells to BNB-endothelial cells, giving rise to the penetration of pathogenic GM-CSF-expressing T/B cells across the BNB. In addition, the present study demonstrated that autocrine secretion of GM-CSF by PnMECs leads to increased permeability of BNB-endothelial cells in typical CIDP.

VCAM-1 is expressed on the surface of inflamed endothelial cells and plays a role in the trans-endothelial infiltration of T cells [26]. In the adhesion and transcellular pathways, T cells adhere to endothelial cells by coupling VLA-4 expressed on T cells with VCAM-1 on endothelial cells [26]. Our previous data demonstrated that the amount of VCAM-1 in PnMECs increased after exposure to MMN-IgG, whereas it was not changed by healthy control IgG, as determined by Western blotting [11]. The present study also showed that the expression of VCAM-1 was increased after exposure to IgG from patients with typical CIDP or MMN in comparison to that in healthy controls. TNF-α is a pro-inflammatory cytokine that upregulates the expression of VCAM-1. Previous study showed that serum concentration of TNF-α was significantly higher in MMN patients than that in ALS patients or patients with other non-inflammatory neurological diseases [27]. The present study demonstrated by immunohistochemistry that TNF-α is expressed by PnMECs after exposure to IgG from patients with MMN, probably inducing the upregulation of VCAM-1 in PnMECs.

The present study is limited by the fact that (1) we were unable to identify the antigen against autoantibodies in IgG from typical CIDP, multifocal CIDP, and MMN, which can bind to PnMECs; (2) we were unable to evaluate the direct transmigration of lymphocytes across the BNB in CIDP and MMN; and (3) we were unable to evaluate the effect of IgG from patients with CIDP and MMN in an in vivo model.

In conclusion, this study demonstrated that GM-CSF and VCAM-1 were increased after exposure to typical CIDP-IgG, and that TNF-α and VCAM-1 were elevated after exposure to IgG from patients with MMN, probably contributing to the infiltration of lymphocytes across the BNB into the PNS in typical CIDP and MMN. The permeability of the PnMECs increased after exposure to IgG from patients with typical CIDP, suggesting breakdown of the BNB induced by IgG. The present study is the first to report that IgG from patients with both CIDP and MMN has a pathogenic effect on BNB-endothelial cells and to identify the underlying molecules responsible for the breakdown of BNB in each disease. These findings indicate that the reduction in serum IgG due to FcRn inhibitor therapy may inhibit the disruption of the BNB stimulated by IgG in both CIDP and MMN. A further analysis is needed to identify novel molecules that bind to BNB.

## 4. Methods

### 4.1. Study Population

This study was approved by the ethics committee of the Medical Faculties of Yamaguchi University (IRB#: #H24-032-6, Approval Date 28 November 2018). Written informed consent was obtained from all the participants. Sera from patients with typical CIDP (n = 15), multifocal CIDP (n = 14), and MMN (n = 12), who were diagnosed at Yamaguchi University Hospital and Chiba University Hospital, were collected. Sera from healthy controls (HCs, n = 14) were also collected. Patients with CIDP or MMN fulfilled the 2021 EAN/PNS diagnostic criteria for CIDP [5] or the 2010 EFNS/PNS diagnostic criteria for MMN [28,29]. Mean age and gender ratio (male–female) at the time of serum sample collection were 48 ± 22 years old, 11:4 in typical CIDP patients, 56 ± 11 years old, 11:3 in multifocal CIDP patients, 45 ± 19 years old, 9:3 in MMN patients, and 38 ± 11 years old, 6:8 in healthy controls. All sera were stored at −80 °C and inactivated at 56 °C for 30 min before the experiments. IgG was purified from the sera using a Melon Gel IgG Spin Purification Kit (Thermo Fisher Scientific, Waltham, MA, USA).

### 4.2. Whole Transcriptome Analyses with RNA-seq

Human PnMECs (FH-BNB cell lines) were used in all the experiments. FH-BNB cells were immortalized with temperature-sensitive SV40 large T antigen (tsA58) and teromease [30].

FH-BNB cells were incubated with IgG from four typical CIDP, four multifocal CIDP, four MMN patients, and four healthy individuals (500 µg/mL) for 12 h at 37 °C. FH-BNB cells that were not incubated with IgG were used as controls.

The method for whole transcriptome analysis with RNA-seq has been previously described [31]. In brief, total RNA was extracted from FH-BNB cells using the RNeasy Mini Kit (Qiagen, Hilden, Germany), and mRNA was purified as described previously [31]. Complementary DNA (cDNA) libraries were produced using a NEBNext Ultra II RNA Library Prep kit (New England Biolabs, Ipswich, MA, USA) and NEBNextplex Oligos for Illumina, as described previously [31]. In this approach, mRNA was fragmented in NEBNext First Strand Synthesis Reaction Buffer at 94 °C for 15 min in the presence of NEBNext Random Primers and was reverse-transcribed with NEBNext Strand Synthesis Enzyme Mix. The library fragments were then concentrated, and index sequences were inserted during PCR amplification. The products were purified using AMPure XP beads (Beckman Coulter, Brea, CA, USA), and the quality of the library was confirmed using an Agilent 2200 TapeStation (D1000, Agilent Thermo Fisher, Waltham, MA, USA). The libraries mixed with equal molecular amounts were sequenced on an Illumina Next-seq DNA sequencer with a 75 bp pair-end cycle sequencing kit (Illumina, San Diego, CA, USA). The data were then trimmed and mapped to the mouse reference genome GRCm38 release-92 using the CLC Genomics Workbench software program (ver. 8.01; Qiagen), as described previously [31]. The mapped read counts were normalized to transcripts per million (TPM) and converted to log2 values after the addition of 1. For the volcano plots, *p* values were calculated using the unpaired Student’s *t*-test, and the fold-change (FC) was determined by subtracting the average values in the HCs from those in the patients. Of the genes with a *p* value < 0.05, those for which the FC increased by >50% or decreased by >50% were used for the Ingenuity Pathway Analysis (IPA), which was performed to analyze the detected genes (Qiagen, Hilden, Germany).

### 4.3. Immunohistochemistry of MIP-3α, Claudin-5, GM-CSF, VCAM-1, TNF-α, ICAM-1, and IP-10 Through the High-Content Imaging Assay

Cells were cultured in MCDB 131 medium containing 500 µg/mL IgG from typical CIDP, multifocal CIDP, and MMN patients or HCs on collagen type 1-coated CELLSTAR^®^ 96-well plates (Greiner, Kremsmünster, Austria) for 24 h. Immunohistochemistry was performed for MIP-3α, claudin-5, GM-CSF, VCAM-1, TNF-α, ICAM-1, and IP-10. Cells were fixed with 4% paraformaldehyde (PFA), permeabilized with 0.3% Triton X-100, and blocked overnight in 5% fetal bovine serum (FBS)/0.3% Triton X-100 in phosphate-buffered saline (PBS) for MIP-3α, GM-CSF, and TNF-α. Cells were fixed with 4% PFA without permeabilization and blocked overnight with 5% FBS in PBS for ICAM-1, VCAM-1, and IP-10. Cells were fixed with 100% ethanol, permeabilized with 1% Triton X-100, and blocked overnight in 5% FBS/0.3% Triton X-100 in PBS for claudin-5.

The cells were incubated with each primary monoclonal antibody (MIP-3α [Novus, Centennial, CO, USA], claudin-5 [Thermo Fisher Scientific, Waltham, MA, USA], GM-CSF [R&D Systems, Minneapolis, MN, USA], VCAM-1 [BD Biosciences, Milpitas, CA, USA], TNF-α [Novus, Centennial, CO, USA], ICAM-1 [Santa Cruz, Santa Cruz, CA, USA], and IP-10 [R&D Systems, Minneapolis, MN, USA]) and then with each secondary antibody (Alexa Fluor 488 anti-rabbit/mouse/goat IgG [Thermo Fisher Scientific, Waltham, MA, USA]).

For high-content imaging [32,33], 5000 cells per well were plated onto CELLSTAR^®^ 96-well plates (Greiner, Kremsmünster, Austria). After immunostaining, the images in the 96-well plate were captured using In Cell Analyzer 2000 (GE Healthcare, Chicago, IL, USA) at ×20 magnification with four fields of view per well (equivalent to almost 800–1000 cells). The images were then analyzed using the IN Carta image analysis software program Version: 1.6.295.8198 (Cytiva, Tokyo, Japan) or the In Cell Analyzer 2000 software program (Cytiva, Tokyo, Japan). The data represent the mean value of six experiments for NF-κB p65 and three experiments for MIP-3α, claudin-5, GM-CSF, VCAM-1, TNF-α, ICAM-1, and IP-10.

### 4.4. Paracellular Permeability of 10 kDa Dextran

FH-BNB cells were cultured on the luminal side, and human peripheral nerve pericytes were maintained on the abluminal side on 0.4 mm pore size 24-well collagen-coated Transwell culture inserts (Corning, NY, USA) for 3 days at 33 °C and then for 2 days at 37 °C [33]. Cells were incubated with 500 μg/mL of individual IgG from patients with t-CIDP (n = 15), multifocal CIDP (n = 14), MMN (n = 12), or HC (n = 14) for 24 h at 37 °C. After the cells were washed, FITC-10 k-Da dextran fluorescence (Sigma-Aldrich, St. Louis, MO, USA) was added to the luminal insert (concentration, 1 mg/mL). A total of 100 μL of medium was then transferred from the abluminal chamber into 96-well black plates for 40 min. Fluorescence signals were measured at 490/520 nm (absorption/emission) using a FlexStation 3 Multi-Mode microplate reader (Molecular Devices, San Jose, CA, USA).

### 4.5. Treatment with GM-CSF Neutralizing Antibodies or GM-CSF

We prepared pooled IgG samples from 10 patients with t-CIDP, 10 patients with multifocal CIDP, and 10 patients with healthy controls. The co-culture BNB in vitro model, composed of FH-BNBs and pericytes, was cultured with IgG from t-CIDP, multifocal CIDP, and HC with a neutralizing antibody (10.0 μg/mL, R&D Systems) against human GM-CSF or normal mouse IgG (control Ab) for 24 h at 37 °C.

### 4.6. Treatment with GM-CSF

Recombinant human GM-CSF (0, 1, 10, and 100 ng/mL, PeproTech, Rocky Hill, NJ, USA) was incubated with FH-BNB for 24 h to assess the permeability of 10 kDa dextran.

### 4.7. Statistical Analyses

All statistical analyses were performed using Prism 7 (Graph Pad ver 9). An unpaired Student’s *t*-test (two-sided) was used for single-comparison analyses. For multiple comparison analyses, a one-way analysis of variance (ANOVA) with Tukey’s multiple comparison test was used. Pearson’s correlation coefficients were used to assess the associations. *p* values of <0.05 and <0.01 were considered to be statistically significant.

## Data Availability

The original contributions presented in this study are included in the article/Supplementary Material. Further inquiries can be directed to the corresponding author.

## Supplementary Materials

The following supporting information can be downloaded at: https://www.mdpi.com/article/10.3390/ijms27021088/s1.
