# Supplementary material for: Blood–Nerve Barrier Breakdown Induced by Immunoglobulin G in Typical and Multifocal Chronic Inflammatory Demyelinating Polyneuropathy and Multifocal Motor Neuropathy
Source: Int J Mol Sci. 2026 Jan 22;27(2):1088. doi: 10.3390/ijms27021088 (PMC12842371; doi:10.3390/ijms27021088)
Supplement: Supplementary file 1 [file ijms-27-01088-s001.zip › ijms-4075537-supplementary.pdf]

Supplemental Figure

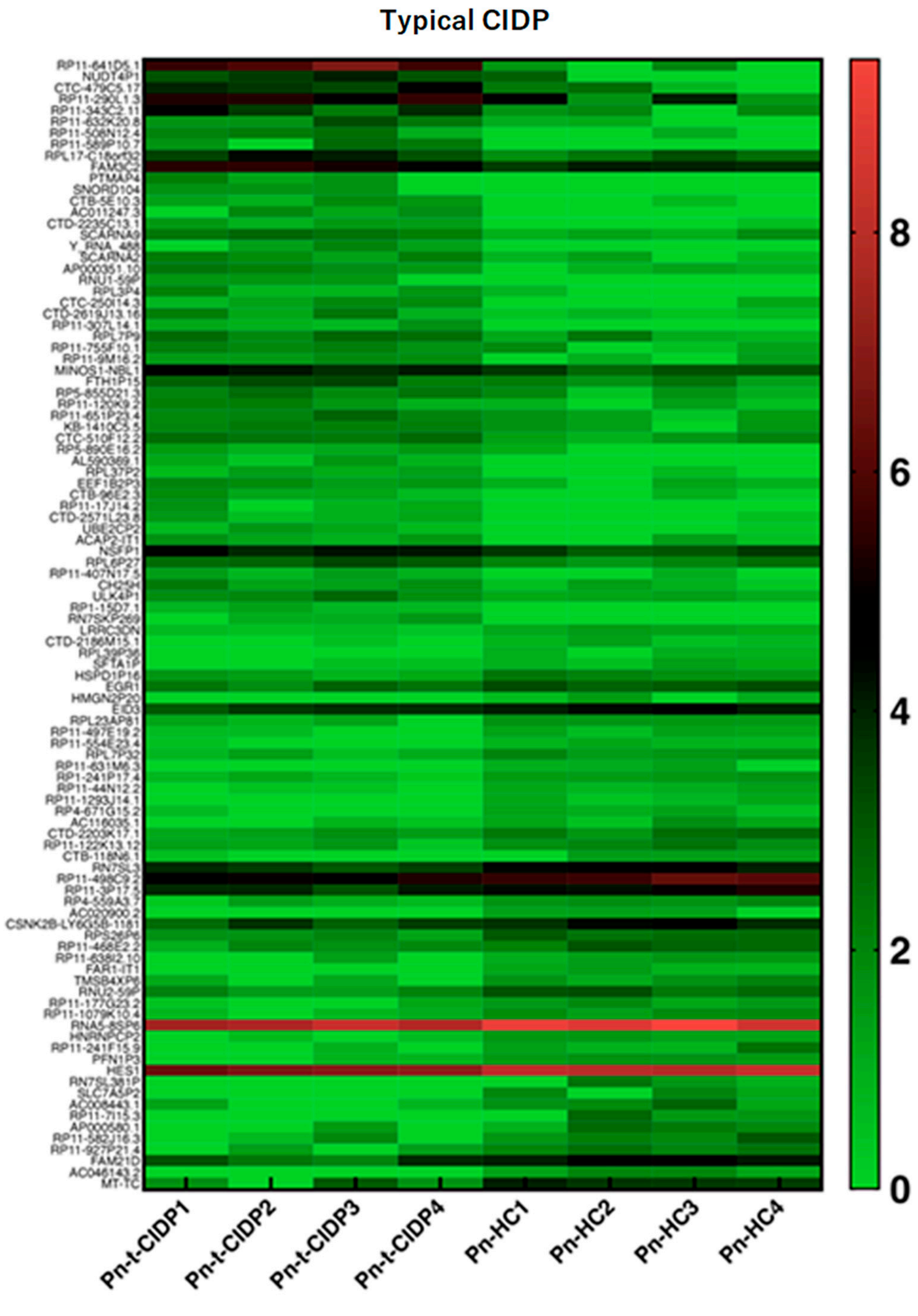

Supplemental Figure S1. Heatmap showing the altered gene expression between typical CIDP and healthy control group

Heat maps revealed that top 100 genes were significantly differentially upregulated (P <0.05) between typical CIDP patients (t-CIDP) (n=4) and healthy control groups (n=4).

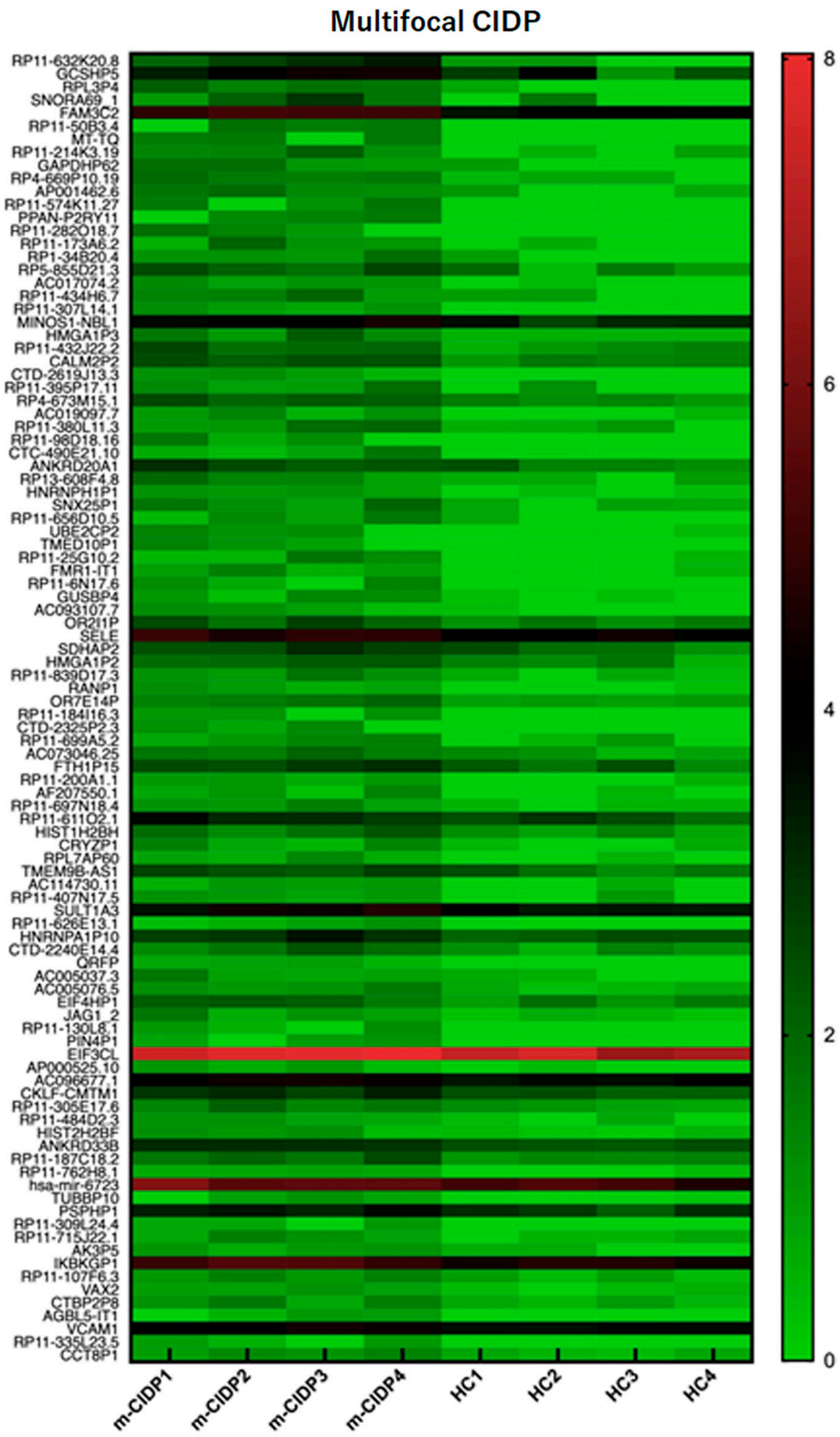

Supplemental Figure S2. Heatmap showing the altered gene expression between multifocal CIDP and healthy control group

Heat maps revealed that top100 genes were significantly differentially upregulated (P <0.05) between multifocal CIDP (m-CIDP) patients (n=4) and healthy control groups (n=4).

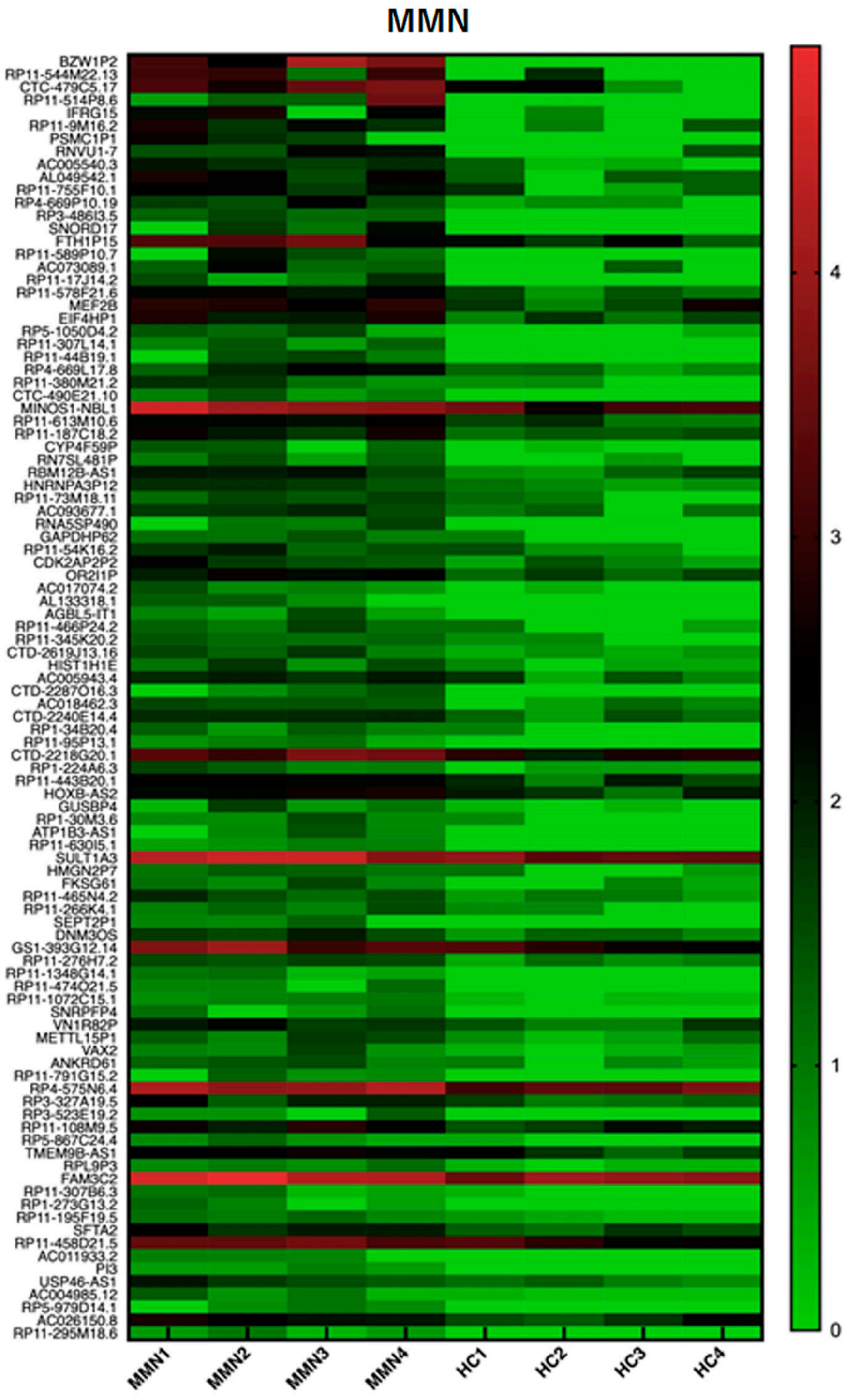

Supplemental Figure S3. Heatmap showing the altered gene expression between MMN and healthy control group

Heat maps revealed that top100 genes were significantly differentially upregulated (P <0.05) between multifocal motor neuropathy (MMN) patients (n=4) and healthy control groups (n=4).

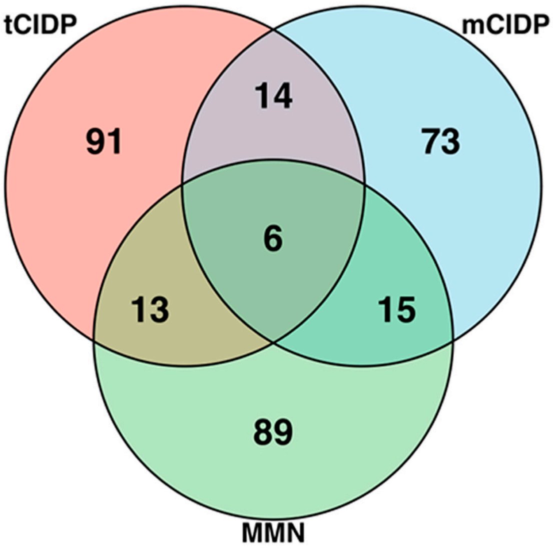

Supplemental Figure S4. Venn diagram showing the intersection of differentially increased expressed genes between the groups.

Venn diagram revealed the intersection of differentially increased expressed genes (FC >1.5; P <0.05) among the typical CIDP (tCIDP), multifocal CIDP (mCIDP) and MMN groups. The common 6 genes were MINOS1-NBL1, RP11-307L14.1, RP1-34B20.4, FTH1P15, CTC-490E21.10 and FAM3C2.

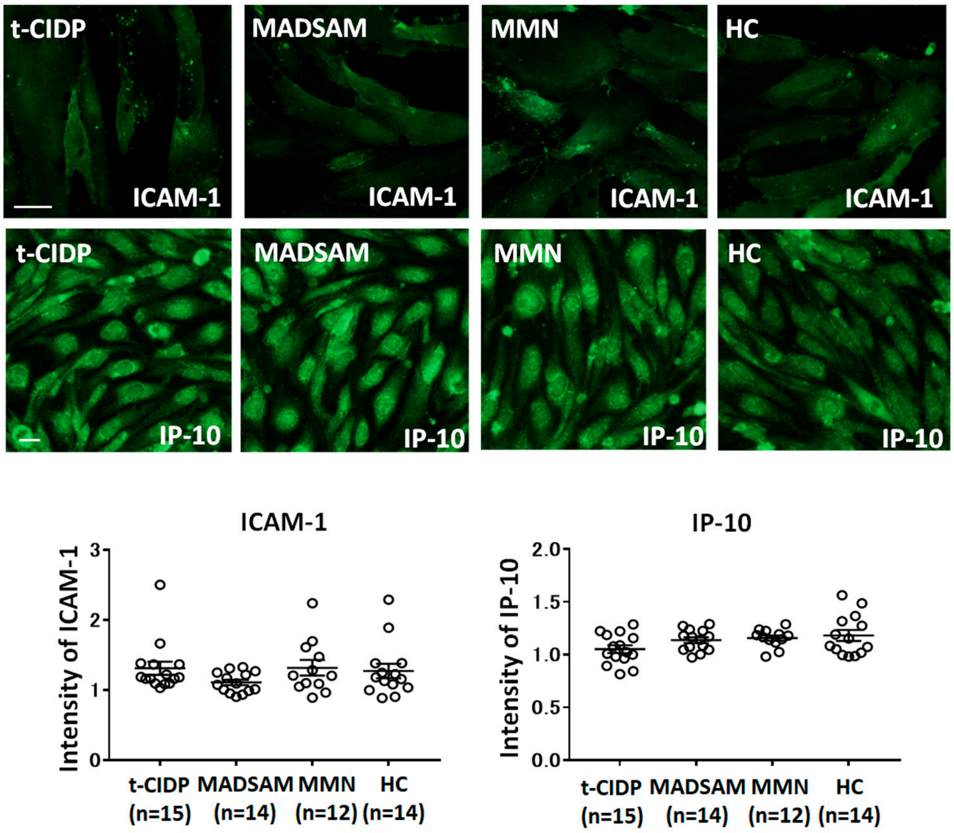

Supplemental Figure S5. Changes of the ICAM-1 and IP-10 after exposure to IgG from patients with typical CIDP, multifocal CIDP and MMN, and healthy controls

Immunostaining of human peripheral nerve microvascular endothelial cells (PnMECs) for ICAM-1 and IP-10 (green) after exposure to IgG (500 μg/mL) from typical CIDP, multifocal CIDP, and MMN patients or healthy con-trols (HC). Images were captured using an in-cell analyzer 2000. Scale bar, 50 μm. Scatter plots of the intensities of ICAM-1 and IP-10 (green) in PnMECs, as determined by high-content imaging after exposure to IgG from patients with typical CIDP (n=15), multifocal CIDP (n=14), MMN (n=12) or healthy controls (HCs, n=14). The data were normalized to cultures that had not been exposed to human IgG, and are shown from three independent experi-ments. P values were determined using an unpaired Student's t-test (two-sided) (*P<0.05, vs. the HC group).

Supplemental Material. List of genes with upregulated or downregulated expression in typical CIDP (t-CIDP), multifocal CIDP (m-CIDP) and MMN

t-CIDP			m-CIDP									MMN		
upregulated		downregulated	upregulated					downregulated				upregulated		downregulated
hsa-mir-6723	RP11-380L11.3	RP11-7O11.3	AC004943.1	RHOT1P2	SUMO2P1	RP11-98D18.16	AL162151.3	AC034102.1	RP11-95I16.4	RP11-421L21.2	AGAP9	FKSG61	RP3-523E19.2	RP11-366L20.3
RP11-206L10.1	RPL18P10	RP4-671G15.2	AC005954.3	HIST1H1B	EEF1A1P16	SETP14	RP11-498P14.3	NICN1-AS1	RP11-138I1.3	RN7SL2	MIR3605	RP11-108M9.5	RP11-474O21.5	RP11-589F5.4
RN7SKP269	BIVM-ERCC5	PFN1P3	RP11-447L10.1	CTD-2538C1.2	RP11-715J22.1	RP11-109L13.1	FABP5P7	GS1-114I9.1	MIR320A	RP11-616K22.1	AP000275.65	SFTA2	RP1-34B20.4	LIMS3_1
MINOS1-NBL1	RP11-407N17.5	RP4-559A3.7	AC007956.1	ANKRD61	RP11-216L13.17	OR2I1P	AP001462.6	RP4-781K5.2	RP11-3P17.5	TERT	RP11-638I2.10	CSF3	TMEM9B-AS1	RP11-750H9.7
RP11-767N6.2	RHOQP1	RP11-1293J14.1	RPS14P3	JAG1_2	RP11-762H8.1	ALG9_2	RP11-787I22.3	MIR4461_2	RP11-338N10.3	RP11-4C20.4	RP13-317D12.3	TUBB8P1	VN1R82P	CTB-25B13.13
RPL7P9	CHURC1-FNTB	HMGN2P20	ACAP2-IT1	RP11-383F6.1	AC005076.5	CALM2P2	TNFSF12-TNFSF1	RP11-360L9.8	AL162407.1	RP11-83J16.1	GPAA1P1	RP11-894P9.1	FAM47E-STBD1_2	RPL12L3
RP11-305E17.6	RPL3P4	HNRNPCP2	BMS1P2	RP11-755F10.3	RN7SL268P	RP11-436G20.1	AC093484.4	RP11-582J16.3	RP11-145E5.5	RP5-886K2.3	MUSTN1	HNRNPA1P10	MINOS1-NBL1	HMGB1P5
SCARNA2	RP11-32B5.1	AC116035.1	RP11-611O2.5	PKD1P1	CTD-2235C13.1	AC019097.7	PPAN-P2RY11	RP11-63M22.2	RN7SL80P	RPL39P36	RHEBP2	ANKRD61	RP11-54K16.2	SMIM2-AS1
SRGAP2C	ULK4P1	MUSTN1	LSM12P1	AC103809.1	RPL7AP60	Z83840.1	RP5-1050D4.4	RP11-19P22.8	AP001505.9	CTB-25B13.13	AC004383.3	AC004985.12	HIST1H1E	C19orf77
RNU1-59P	RP11-632K20.8	RP11-797D24.4	AC006449.1	IKBKGP1	PSPHP1	HNRNPH1P1	RP11-1035H13.3	CTD-2369P2.12	HIST1H4K	RP11-62F24.2	RNU6-516P	DNM3OS	HMGN2P7	SERF1A
NUDT4P1	RP11-120K9.2	RP11-3P17.5	AC007401.2	HIST1H2BH	RP1-206D15.5	RP11-680G10.1	PSMC1P1	Y_RNA_548	RP11-169K16.4	RN7SL784P	RP11-264M12.2	RP11-145M9.5	CTD-2287O16.3	hsa-mir-1199
AC096677.1	RP11-97O12.7	HES1	CXCL2	HIST2H2BD	RPLP1P11	RP11-669E14.6	HIST2H2BC	DHRS4-AS1_1	RP11-462G2.1	AC117395.1	RP11-411B10.3	RP11-443B20.1	RP5-1050D4.2	AC004980.9
RP11-134P9.3	RP11-304L19.11	AC046143.2	RP5-1164C1.2	AF207550.1	AGBL5-IT1	RP11-589P10.7	RP11-61N20.3	ATP6V1G2-DDX39B	DPH3P1	AL590822.2	AC010468.1	AC018462.3	GAPDHP62	IL10RB-AS1
RP11-95P13.1	NPIPA5	RP11-496H1.2	RP11-467L19.8	AC174470.1	RPL34P18	AC073046.25	RP11-707G14.1	AC046143.2	C17orf100	RPSAP58	RP11-567L7.5	AC011933.2	RP11-466P24.2	RPL5P34
RP11-443B20.1	EIF3CL	RP11-241F15.9	RN7SL844P	RP11-247L20.4	PIK3R2_2	CTD-3193K9.3	RP11-350N15.6	RP11-254F7.3	Y_RNA_47	RP11-425D10.1	AC009492.1	FAM3C2	HNRNPA3P12	AP000254.8
AC011247.3	CKLF-CMTM1	CTD-2186M15.1	RN7SL481P	RP3-473L9.4	ANKRD20A1	AC011330.5	AC010733.5	CEMP1	MT-TY	RP11-10G12.1	RP13-1039J1.4	AC026150.8	OR2I1P	SNX18P13
RP11-301O19.1	CTC-479C5.17	CTD-2203K17.1	RP11-490H24.5	RN7SL602P	QRFP	RP11-307L14.1	RP11-290L1.3	MT-TC	AL050335.1	RP5-884G6.2	AC037459.4	SEPT2P1	RP11-187C18.2	NUDT17
GPR17	RP11-343C2.11	RP11-631M6.3	RP11-20B24.7	SNORD100	TNFRSF13C	RP11-51F16.9	RP11-514P8.6	FAM21D	AL672183.2	RNU6-510P	OR7E13P	RP1-155D22.2	RP11-345K20.2	RP11-543C4.1
RP11-33O4.1	AC009120.5	AC020900.2	GOLGA6L9	SELE	RP11-466P24.2	RP11-656D10.5	RP11-214K3.19	AL139385.1	RP3-486I3.4	CGA	RP11-677M14.6	VAX2	RP11-266K4.1	RP11-4C20.4
CCL20	RN7SL105P	CTB-118N6.1	SNORD17	C17orf67	RP3-486I3.5	CTD-2619J13.3	RP11-50B3.4	RPS26P6	CTC-513N18.7	RP11-651P23.4	SSSCA1-AS1	TAS2R19	CTC-490E21.10	RP11-411B10.4
AC097635.4	GP1BA	MTND6P4	LTB	VCAM1	TMEM9B-AS1	RP11-330M2.4	CTC-429P9.4	MT-TL1	U3_13	RPL7P1	RP11-322E11.6	RP11-407G23.4	RP11-44B19.1	RP11-523H24.3
AC098614.2	RP11-589P10.7	EGR1	CTGLF12P	SLC7A5P2	RANP1	UBE2CP2	ATP5G1P4	RP11-927P21.4	RP11-241F15.9	CTC-301O7.4	RPS26P3	SOGA3_1	CYP4F59P	RP11-967K21.1
FCF1P2	CTB-96E2.3	AC008443.1	RPS11P5	RP11-187C18.2	EIF4HP1	RP4-541C22.5	RAB43P1	AC008865.1	CTC-471J1.8	CTD-3220F14.2	AC144568.4	USP46-AS1	RNA5SP490	RP11-722E23.2
RP11-3B7.1	RP11-848P1.7	RPL15P3	SNORA7B	RP5-1180E21.5	AC090587.4	AL583842.3	BZW1P2	RP11-316M1.12	C1orf134	RP11-797D24.4	RP11-391M20.1	PHOSPHO1	RP11-380M21.2	CTA-313A17.3
RP11-755B10.4	NSFP1	HIST1H3F	USP46-AS1	BX088651.1	HIST2H2BF	RP11-21A7A.3	RPL3P4	AC004837.3	RP11-629O1.2	RPS15AP10	RP11-376P6.3	RP11-154P18.2	RP4-669P10.19	IFNWP19
RP11-651P23.4	RP5-890E16.2	CSNK2B-LY6G5E	hsa-mir-6723	RP11-6N17.6	RP11-242D8.1	AC073089.1	MSANTD3-TMEFF	CTC-564N23.2	RN7SL737P	AC005606.15	snoU13_283	PI3	HOXB-AS2	RPS4XP13
RP11-432B6.3	CTD-2510F5.4	CYCSP55	ITPR1-AS1	ULK4P2	RPS20P33	SIGLEC14	RP11-611O2.3	RP11-468E2.2	RN7SL381P	AC108463.1	AC016700.6	CTD-2240E14.4	RP11-578F21.6	C10orf35
RP11-641D5.1	SNORD104	RP11-177G23.2	RP11-603J24.7	RPL17-C18orf3	RP11-626E13.1	MT-TQ	SNORA69_1	BLOC1S5-TXNDC5	RP11-227B21.2	RP11-598P20.5	RP11-404P21.9	RP11-465N4.2	RP11-307L14.1	AC097523.1
ACAP2-IT1	RP11-649A18.4	RP3-399J4.2	RP13-401N8.3	RBM12B-AS1	HNRNPA1P10	CTB-96E2.3	RP11-45M22.4	ST20-MTHFS	HIST2H2AC	HSPE1-MOB4	RN7SKP11	CSPG4P10	RP11-613M10.6	AP000936.1
RP11-338K13.1	RP11-806L2.2	HSPD1P16	RP11-388C12.5	RP11-51F16.5	RP11-136K14.1	RN7SL417P	GCSHP5	RPPH1	RP5-994D16.9	PPIAP9	RP11-379F4.7	RP5-979D14.1	RP11-73M18.11	CTC-471J1.8
RP11-241F15.1	RPL6P27	RPL7P32	AC007387.2	RP11-613M10.6	RP11-310E22.4	FAM115D	NPFF	AL392111.1	RP11-514P8.8	HIST2H4A	RP11-649E7.5	RP1-224A6.3	MEF2B	FTH1P1
TMSB4XP8	UBE2CP2	RP11-180C16.1	RPS14P4	CKLF-CMTM1	BTF3L4P2	Y_RNA_582	RP11-632K20.8	AL034548.1	RP11-806L2.6	RP11-809N8.5	RP13-152O15.5	RP1-273G13.2	EIF4HP1	HIST1H3F
RP11-307L14.1	RP11-17J14.2	RP11-582J16.3	CTC-429P9.1	AC005037.3	HIST2H4B	RP11-575L7.8	RAB4B-EGLN2	RP11-180C16.1	RP11-861E21.1	RP11-290L1.4	RP11-680A11.5	RP11-192H23.6	AC093677.1	FAM47E-STBD1_
CTD-2235C13.1	RPL17-C18orf32	RP11-44N12.2	RP11-157J24.2	FTH1P15	RP11-452K12.4	RP11-178L8.4		MT-TS1	RP11-4C20.3	SNORA76_3	AC005523.2	SULT1A3	RP4-669L17.8	FAR1-IT1
RP1-90J20.8	CTC-510F12.2	RPS26P6	CTBP2P8	RP11-632F7.1	CTD-2240E14.4	AC017074.2		CTD-2278I10.6	RNA5SP393	RP11-299M14.2	GOLGA8K	RP11-195F19.5	RP11-17J14.2	RN7SL834P
AL583828.1	CTC-250I14.3	SFTA1P	RP11-335L23.5	RP11-184I16.3	RP5-855D21.3	LINC01040		CTA-313A17.3	MTCP1	OGFR-AS1	USP12-AS2	RP11-295M18.6	PSMC1P1	RP11-613M5.2
RP1-34B20.4	CTB-5E10.3	FAM21D	RP11-385F7.1	AK3P5	TMED10P1	RP11-574K11.27		RP4-631H13.6	RN7SL1	RP11-316M21.7	RP1-59D14.1	CTD-2218G20.1	AL049542.1	RN7SL525P
HIST1H2BH	LLNLR-246C6.1	RNU2-59P	RP11-443B7.3	LINC00659	SULT1A3	RP13-608F4.8		RP11-488C13.7	RPL23AP81	Z73979.1		RP4-575N6.4	RP11-589P10.7	SFTA1P
RP1-15D7.1	CTD-2528A14.5	RP11-122K13.12	CSF2	RP11-197P3.5	GUSBP4	HMGN2P24		AC068831.15	TECRP1	RP11-177G23.2		RPL9P3	RP3-486I3.5	EGFLAM-AS1
FTH1P15	CTD-2527I21.9	FAR1-IT1	KCNJ6-IT1	AC018804.6	RP11-200A1.1	RP4-669P10.19		PPIAP29	TIMM8AP1	RP1-241P17.4		RP1-292B18.1	AC005540.3	SNORA70_5
RP3-486I3.5	AC104534.2	AP000580.1	AC100830.4	RP11-699A5.2	AC005540.3	RN7SL608P		SNRPEP4	AP000487.6	RN7SKP80		OSTCP8	SNORD17	RP11-212P7.1
RP5-1165K10.2	CTC-490E21.10	RP11-7I15.3	GTF2IP1	SNORD104	Y_RNA_752	RP11-508N12.4		CTD-2218G20.1	RN7SL735P	LBX2		RP3-327A19.5	RP11-755F10.1	SNORA65
VN1R42P	CTD-2571L23.8	RP11-254B13.1	RP11-10A14.3	RPL12P4	RP3-402G11.28	RPL21P119		RN7SL529P	AP006216.10	GOLGA6L20		RP11-307B6.3	RNVU1-7	RP1-257A7.5
AC005102.1	CTD-2619J13.16	RP11-554E23.4	ST13P6	NUDT4P1	RP11-755F10.1	AC093162.3		CXCR5	RP1-20C7.6	PARD3-AS1		RP5-867C24.4	RP11-9M16.2	CTAGE8
ZC3HAV1L	RP4-800J21.3	EID3	AC010761.9	EEF1B2P3	RP11-380L11.3	SHPK_1		RP11-135L13.4	RP11-104G3.2	BX005214.1		RP11-1072C15.1	IFRG15	RPS26P3
RP5-855D21.3	AP000351.10	RP11-468E2.2	SNRPGP4	HMGA1P2	RP5-890E16.2	7SK_3		AC008443.1	RP11-318K12.3	HES1		RP11-95P13.1	RP11-514P8.6	AL392111.1
POMK	LINC00685	RP11-649E7.5	DNM3OS	ANKRD33B	RP11-126L15.4	RP11-282O18.7		AL645728.2	BCL2L2-PABPN	SNX18P13		AC005943.4	CTC-479C5.17	RP11-91G21.1
RBM12B-AS1	FAM3C2	RN7SL3	AL359973.1	RP11-361L15.4	AC093107.7	AP000640.10		AL158040.1	RP11-245J9.6	Metazoa_SRP_11		RP11-304L19.11	RP11-544M22.13	RP11-582J16.3
KB-1410C5.5	EEF1B2P3	AL163953.3	RP11-407N17.5	AC114730.11	RP11-395P17.1	AC018462.3		CSNK2B-LY6G5B-1	HIST1H3F	AC093807.1		SNRPFP4	BZW1P2	RN7SL809P
RP11-128L5.1	IKBKGP1	RP11-497E19.2	SYNJ2BP-COX1	CTC-250I14.3	RP11-461A8.4	XX-C2158C6.2		RP11-498C9.16	TSACC	SNORD97		RP11-1348G14.1		RP11-180C16.1
RP11-195F19.5		RP11-638I2.10	RP11-227G15.2	RP11-672A2.5	NBPF2P	MINOS1-NBL1		RP11-402D21.2	RPSAP14	RPL7AP6		AL133318.1		AC015987.2
RP11-112J3.16		CTD-2651B20.1	AC092570.3	RPL12P1	AC092597.3	RP11-1020A11.1		HNRNPCP2	RP11-932O9.7	RP4-559A3.7		RP11-276H7.2		RP11-282O18.6
CR848007.2		CTD-3110H11.1	RP11-855A2.3	MRPS17_1	AP000580.1	RP11-574K11.31		NPIPA7_2	RP11-12M9.3	LINC01063		RP13-39P12.2		RP11-7I15.3
RP11-250H24.6		SLC7A5P2	RP11-309L24.4	CTC-471J1.10	RP11-697N18.4	CTD-2619J13.16		CTB-60E11.4	SNORA70_5	CTD-2616J11.11		AGBL5-IT1		RP11-19P22.8
RP11-508N12.4		RP11-249C24.10	RP11-864I4.3	TRAV13-2	AC004878.3	COMMD3-BMI1		PFN1P3	PYY2	DBIL5P		METTL15P1		PTGER4P2
RP11-9M16.2		RN7SL381P	MIR635	RP11-305E17.6	HMGN2P15	RP11-155O18.6		AC114546.1	CTD-2203K17.1	AC007952.6		RBM12B-AS1		DHRS4-AS1_1
AL590369.1		RP11-368I7.4	RP11-512M8.5	CRYZP1	RP11-54F2.1	RP1-34B20.4		RNA5-8SP6	HMGN2P8	HERC2P7		CTD-2270L9.4		RP13-608F4.5
RP11-216L13.17		DBIL5P	RP11-130L8.1	PIN4P1	TUBBP10	RP11-25G10.2		CTD-2192J16.22	RP4-647C14.3	RP11-563H6.1		ATP1B3-AS1		CTC-564N23.2
RP11-499P20.2		RP11-1079K10.4	RP11-107F6.3	RP1-224A6.3	RP4-673M15.1	AC092798.2		RP11-761N21.2	CTA-14H9.5	RP11-7O11.3		CLLU1OS		FAM21D
CH25H		RP11-927P21.4	AL662890.3	AGAP4	HMGB1P3	HMGA1P3		RP11-7I15.3	AC068987.1	MYZAP		RP11-630I5.1		RP11-63M22.2
RP11-162A23.5		TEN1-CDK3	RP11-484D2.3	SNX25P1	SDHAP2	RP11-9M16.2		ATP5EP2	RP11-119B16.2	RP11-332H14.1		RP11-791G15.2		
OR7E14P		RP11-498C9.2	RP11-507E23.1	RNY3P8	CTC-490E21.10	RNU4-52P		RP11-337C18.10	RP11-712L6.5	RP11-281O15.7		RP11-288L9.4		
Y_RNA_488		RP11-498C9.3	EIF1AXP1	LGALS7B	OR7E14P	RP11-173A6.2		AC119673.1	RP11-466H18.1	RP1-30G7.2		AC073089.1		
RN7SL23P		RP13-270P17.2	RP11-95P13.1	RP11-785H5.1	LA16c-390E6.4	SNORD3C		RP11-109P14.10	RP11-305K5.1	RN7SL15P		CDK2AP2P2		
RP11-755F10.1		CTB-25B13.13	EFCC1	VAX2	AJ271736.10	C16orf98		AC015987.2	RP3-399J4.2	RPL15P3		RP1-30M3.6		
RPL37P2		RPSAP58	AL365361.1	HIST1H3E	RP11-732M18.3	AMIGO3		RN7SL3	RP11-1072C15.	RP11-78A19.3		CTD-2619J13.16		
RP11-320L11.2		RPL39P36	RPS26P8	CCT8P1	RP11-839D17.3	GAPDHP62		XXbac-BPGBPG55C	SUMO1P3	ALG1L15P		SRSF9P1		
SCARNA9		RPL23AP81	RPL6P27	RP5-1052M9.1	RP11-432J22.2	RP11-762I7.5		RP11-554J4.1	RP11-175I17.2	RP11-426L16.3		GS1-393G12.14		
RP11-320N7.1		TMSB4XP6	RP11-295P9.12	EIF3CL	RP11-347C12.1	RP11-242D8.3		RP11-517A5.5	RP11-326C3.7	AP003419.11		IGKV1OR-2		
RP5-940J5.9		LRRC3DN	AC004917.1	SNORD116-20	SCARNA7	RP11-434H6.7		RP11-770J1.5	HIST1H2BPS2	RPL18AP7		GUSBP4		
PTMAP4		Z73979.1	PRELID1P4	AP000525.10	CTD-2325P2.3	RP5-977B1.11		RP11-697E22.1	AC104651.1	RP11-54C4.1		AC017074.2		
RP11-977G19.5		RP1-241P17.4	RP11-611O2.1	AC096677.1	FMR1-IT1	AC091738.2		RP1-43E13.2	RP11-411B10.4	CTD-2002J20.1		RP11-458D21.5		
RP11-290L1.3		RNA5-8SP6	HMGA1P8	RP11-304L19.4	SEPT2P1	FAM3C2		RNU2-2P	RP11-89C3.4	AC061975.1		RN7SL481P		
CLLU1OS		MT-TC	AC112719.1	LL0XNC01-237I	CDRT4	CTA-253N17.1		RN7SL525P	CTD-2210P24.4	AC108479.2		FTH1P15		
